# Comparing whole-genome sequencing to pulsed-field gel electrophoresis for vancomycin-resistant *Enterococcus faecium* hospital surveillance

**DOI:** 10.1128/spectrum.00847-26

**Published:** 2026-05-26

**Authors:** Joshua Mayoral, Michael Malczynski, Javier Ruiz, Wanda Polanco, Kevin Fritz, Jovanneé Nunez, Shardul N. Rathod, Kendall Kling, Chao Qi

**Affiliations:** 1 Department of Pathology, Northwestern University Feinberg School of Medicine, Chicago, Illinois, USA; 2 Clinical Microbiology Laboratory, Northwestern Memorial Hospital, Chicago, Illinois, USA; 3 Department of Healthcare Epidemiology and Infection Prevention, Northwestern Memorial Hospital, Chicago, Illinois, USA; 4 Department of Medicine, Division of Infectious Diseases, Northwestern University Feinberg School of Medicine, Chicago, Illinois, USA; Maryland Department of Health, Baltimore, Maryland, USA

**Keywords:** strain typing, outbreak, infection prevention, pulsed-field gel electrophoresis, whole-genome sequencing, vancomycin-resistant enterococci

## Abstract

Vancomycin-resistant enterococci (VREs) have emerged as an important public health concern and a leading cause of healthcare-associated infections. Accurately distinguishing between closely related VRE strains is imperative for infection control measures in the setting of hospital outbreaks. Although pulsed-field gel electrophoresis (PFGE) is considered the gold standard for VRE strain typing, recent studies have identified whole-genome sequencing (WGS) as a promising alternative with greater resolution. In this study, we report on the validation and utility of a WGS and single-nucleotide polymorphism (SNP) analysis pipeline toward vancomycin-resistant *Enterococcus faecium* (VREfm) strain typing in a large academic medical center. Using PFGE as the reference method, we identified a conservative threshold of ≤10 SNPs as robustly identifying related VREfm isolates with 98.5% sensitivity and 93.1% specificity. Comparing VREfm infection control surveillance pre- and post-WGS SNP strain typing revealed a proportionally greater number of isolates with related strain types and epidemiological links in the post-WGS SNP group. These findings suggest that WGS SNP analysis can improve the resolution of emerging hospital outbreaks and infection control measures, as compared to PFGE.

**IMPORTANCE:**

Distinguishing between closely related antibiotic-resistant bacteria is an important task toward infection control measures in a hospital environment. This study focused on strains of one particular antibiotic-resistant bacterium (vancomycin-resistant *Enterococcus faecium*) and assessed the utility of a whole-genome sequencing analysis pipeline toward distinguishing strain relatedness compared to the gold standard reference method. The results showed that strain typing with whole-genome sequencing identified a proportionally greater number of transmission/outbreak events compared to the reference method over a similar time period. The findings suggest that a whole-genome sequencing pipeline, when available, can improve hospital infection control measures.

## INTRODUCTION

Vancomycin-resistant enterococci (VREs), represented predominantly by the species *Enterococcus faecium* (vancomycin-resistant *Enterococcus faecium* [VREfm]), are a leading cause of hospital-acquired infections (1, 2) and are classified as high priority by the 2024 World Health Organization Bacterial Priority Pathogens List (3). The spread of VRE in a hospital setting can be curtailed by the timely identification of outbreaks and subsequent infection prevention measures (4, 5). To reliably detect outbreaks involving VRE strains, strain typing is essential for differentiating true nosocomial transmission events from unrelated, sporadic infections. Several molecular typing strategies for VRE have been refined, demonstrating strong utility for identifying hospital-associated transmission pathways (5–8). It is clear from these recent efforts that whole-genome sequencing (WGS) has emerged as a powerful approach toward high-resolution and accurate strain typing by leveraging single-nucleotide polymorphisms (SNPs) between VRE genomes as indicative of strain relatedness (9–12). However, the widespread adoption of a WGS SNP analysis is curtailed by cost and, in particular, complexity, as the entire WGS SNP pipeline involves sample preparation procedures and bioinformatic analyses with many critical parameters (13).

In this study, we sought to establish a reproducible WGS SNP pipeline and compare the results to those obtained by pulsed-field gel electrophoresis (PFGE), the standard method of choice for VRE strain typing in our clinical microbiology laboratory. Our results indicate that WGS SNP performed comparably to PFGE with respect to sensitivity while also improving the identification of epidemiological links among related VREfm isolates.

## MATERIALS AND METHODS

### Vancomycin-resistant *Enterococcus faecium* isolation

VREfm isolates were obtained between September 2022 and November 2025 from rectal ESwabs (Copan) of patients admitted to select high-risk units of Northwestern Memorial Hospital and Prentice Women’s Hospital. Screening was performed for each patient either on admission, weekly, or as part of a discharge fecal carriage surveillance program. Screening ESwabs were inoculated onto Spectra VRE medium plates (Thermo Fisher) using an automated Walk-Away Specimen Processor instrument (Copan) and incubated at 35°C–37°C for 18 hours. Positive colonies were sub-cultured onto non-selective blood agar plates for biochemical testing, vancomycin resistance confirmation testing by Kirby-Bauer vancomycin disk diffusion (Hardy Diagnostics), and species confirmation by matrix-assisted laser desorption ionization-time of flight mass spectrometry using a Vitek MS instrument (bioMérieux). Confirmed VREfm sub-cultures were then selected for further strain typing as described below.

### Pulsed-field gel electrophoresis

Colonies of confirmed VREfm isolates were used to make optical density suspensions of 1.8–2.0 in Tris-EDTA (TE) buffer. One milliliter of the cell suspension was centrifuged for 5 minutes; the supernatant was decanted; and the pellet was resuspended in 170 µL of a lysozyme (30 mg/mL, Sigma) and mutanolysin (1 U/µL, Sigma) solution. The resuspension was incubated for 25 minutes at 33°C–37°C, after which Proteinase K (50 mg/mL, Epicenter) was added and allowed to incubate at room temperature for 5 minutes to form a plug. Plugs were washed in TE buffer and stored in 2°C–8°C. At the time of electrophoresis, plugs were incubated in 250 µL SmaI restriction enzyme (20 U/µL, New England Biolabs) with CutSmart buffer (New England Biolabs) for 2 hours at 25°C. One percent agarose gels were prepared using Certified Megabase Agarose (Bio-Rad) in 1× Tris-borate-EDTA buffer. The digested samples were loaded in the agarose gel, and electrophoresis was conducted in 1× Tris-borate-EDTA buffer with a GenePath electrophoresis system (Bio-Rad). Agarose gels were stained with SYBR Green I (Thermo Fisher).

Strain comparisons via PFGE were performed within a rolling 6-week period based on the average patient length of stay from hospital units where VRE surveillance screening was in place. As per previously established VRE PFGE strain typing criteria (14), restriction digestion patterns yielding zero to three band differences between two isolates were classified as “closely related,” whereas strains yielding greater than six differences were classified as “distinct.” Strains with four to six band differences were given an intermediate classification of “possibly related.”

### Whole-genome sequencing sample preparation

*E. faecium* DNA was extracted using the automated BD Max system (Becton Dickinson) from inoculated tryptic soy broth cultures grown for 18–24 hours at 37°C. Sample libraries were prepared from 100 to 500 ng of DNA using the Illumina DNA Prep Kit and barcoded using the Illumina DNA/RNA UD Indexes Set A Kit per manufacturer instructions. Library concentrations and fragment sizes were assessed with Qubit and Agilent 5300 Fragment Analyzer instruments, respectively. Sample libraries were normalized by diluting to a concentration of 4 nM, pooled, and denatured in 0.2 N NaOH. The pooled denatured libraries were diluted to a final loading concentration of 20 pM. PhiX run control (Illumina, FC-110-3001) was diluted, denatured, and loaded as described for the sample libraries. Up to 18 sample libraries were run on an Illumina MiSeq System using the MiSeq Reagent Kit v3 150 Cycle Cartridge (MS-102-3001) with paired-end reads.

### Sequence data analysis

FASTQ data files collected from MiSeq sample runs were imported into CLC Genomics Workbench (Qiagen) software (v24.0.1, settings described in Table S1). For each run, quality metrics were obtained; adapters and barcodes were trimmed; and isolate genomes were assembled via reference-based assembly. To construct a K-mer tree comparing reference genomes to a subset of isolates, the “Create K-mer tree” tool was used in the CLC Genomics Workbench (K-mer length of 25) together with three complete genomes, each from *E. faecium* clades A1, A2, and B, selected from a previously compiled list of *E. faecium* strains (15). The *E. faecium* strain NRRL B-2354 (NCBI GenBank accession number CP004063.1, genome assembly ASM33640v1, assembly ID GCA_000336405.1) was selected as the reference genome for all reference-based genome assemblies on the basis of MLST typing results obtained via the Bezdicek et al. (16) *E. faecium* MLST scheme in the CLC Genomics Module “Type with MLST Scheme.” Based on minimal WGS quality metric value criteria post-reference genome assembly, the consensus sequences for isolates of sufficient quality were aligned to identify SNPs (workflow “Compare Variants Across Samples”) and construct SNP trees using the “Neighbor Joining Tree Construction” algorithm. Post-WGS SNP validation (beginning 11 November 2024), a rolling 6-week period was used for strain comparisons based on the average length of stay of patients on wards undergoing VRE surveillance at that time.

### Hospital VRE epidemiological investigations

VRE transmission review was performed on a weekly basis by our hospital's infection prevention team using strain typing results obtained from either PFGE (up to 10 November 2024) or WGS SNP (beginning 11 November 2024). Patients with matching strain type designations (defined as closely related or possibly related) were reviewed for epidemiological links, defined as inpatient geo-temporal overlap (patients admitted to the same unit at the same time), personnel overlap (patients on different units but cared for by the same healthcare providers), and/or procedural overlap (patients in the same procedural area, e.g., operating room and interventional radiology suite). A transmission was defined as two patients with matching strain types and at least one epidemiological link within the PFGE or WGS rolling window. An outbreak was defined as three or more transmissions involving the same strain type attributed to the same inpatient unit within a 4-week period.

### Statistical analyses

All statistical analyses, tables, and graphs were prepared using R statistical software version 4.2 (17) with the R packages ggplot2 (18) and gt (19).

## RESULTS

### Establishing a WGS SNP pipeline for VRE strain typing

VREfm isolates collected during the course of routine hospital VRE surveillance from September 2022 to November 2024 were used for WGS SNP pipeline validation. During this period, PFGE migration patterns were assessed between isolates within a 6-week period as part of the standard strain typing workflow in our laboratory, and patients with closely related or possibly related comparisons to other PFGE strain types triggered transmission investigations by our hospital infection prevention team. A total of 58 unique VREfm isolates with predetermined PFGE strain type designations were selected for WGS SNP validation.

**Fig 1:**
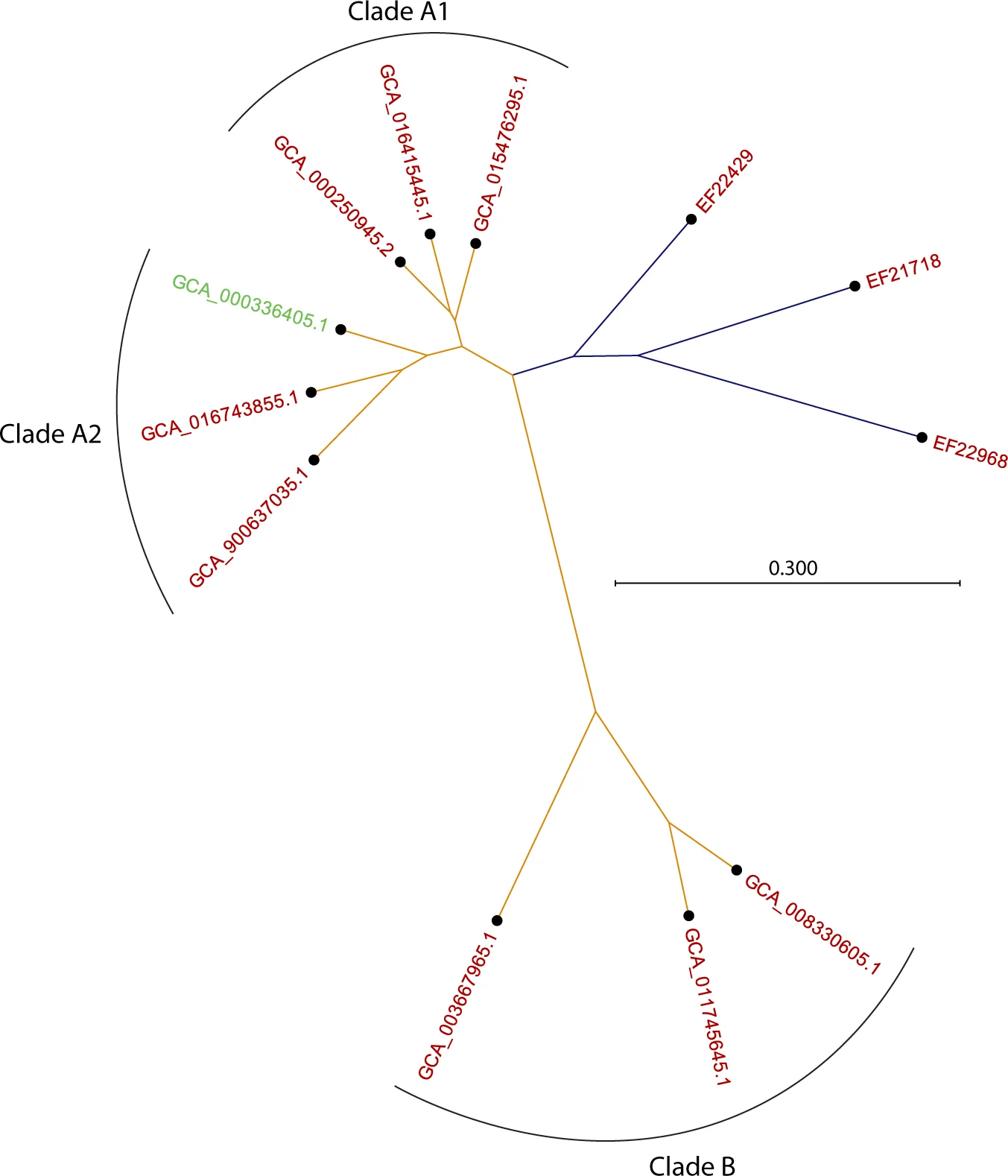
K-mer tree of VREfm isolates of this study and representative *E. faceium* genomes. A K-mer tree depicting the phylogenetic relationship between a sub-sample of VREfm isolates of this study (“EF,” blue branches) and three complete genomes each from the three previously described *E. faecium* clades: A1, A2, and B (“GCA,” orange branches). K-mers of length 25, identified from either trimmed reads of isolates or the complete *E. faecium* reference genomes, were used to construct the tree. The scale bar represents the K-mer distance between genomes based on Jensen-Shannon divergence, as calculated within the CLC Genomics Workbench software. The isolates of this study are closer in distance to Clades A1 and A2, as opposed to Clade B. The *E. faecium* reference strain selected for reference-based genome assemblies is highlighted in green text (GCA_000336405.1).

Given that our pipeline centered on strain-level differences among VREfm isolates within a 6-week period, we opted for a reference-based genome assembly strategy in the WGS SNP analysis pipeline. A K-mer tree was constructed using an initial sub-sample of three isolates and a sample of nine complete genomes from *E. faecium* reference strains belonging to Clades A1, A2, and B (Fig. 1). Inspection of the resulting K-mer tree revealed that the initial set of isolates exhibited closer relatedness to Clades A1 and A2 compared to Clade B, noting a minimal K-mer distance separated the isolates from Clades A1 and A2 (Fig. 1). Strain NRRL B-2354 (Fig. 1, green accession ID) was selected for all subsequent reference-based genome assemblies based on typing results obtained in the CLC Genomics Workbench software using the Bezdicek et al. *E. faecium* MLST typing scheme (16).

### Quality control and reproducibility of the WGS SNP pipeline

**Fig 2:**
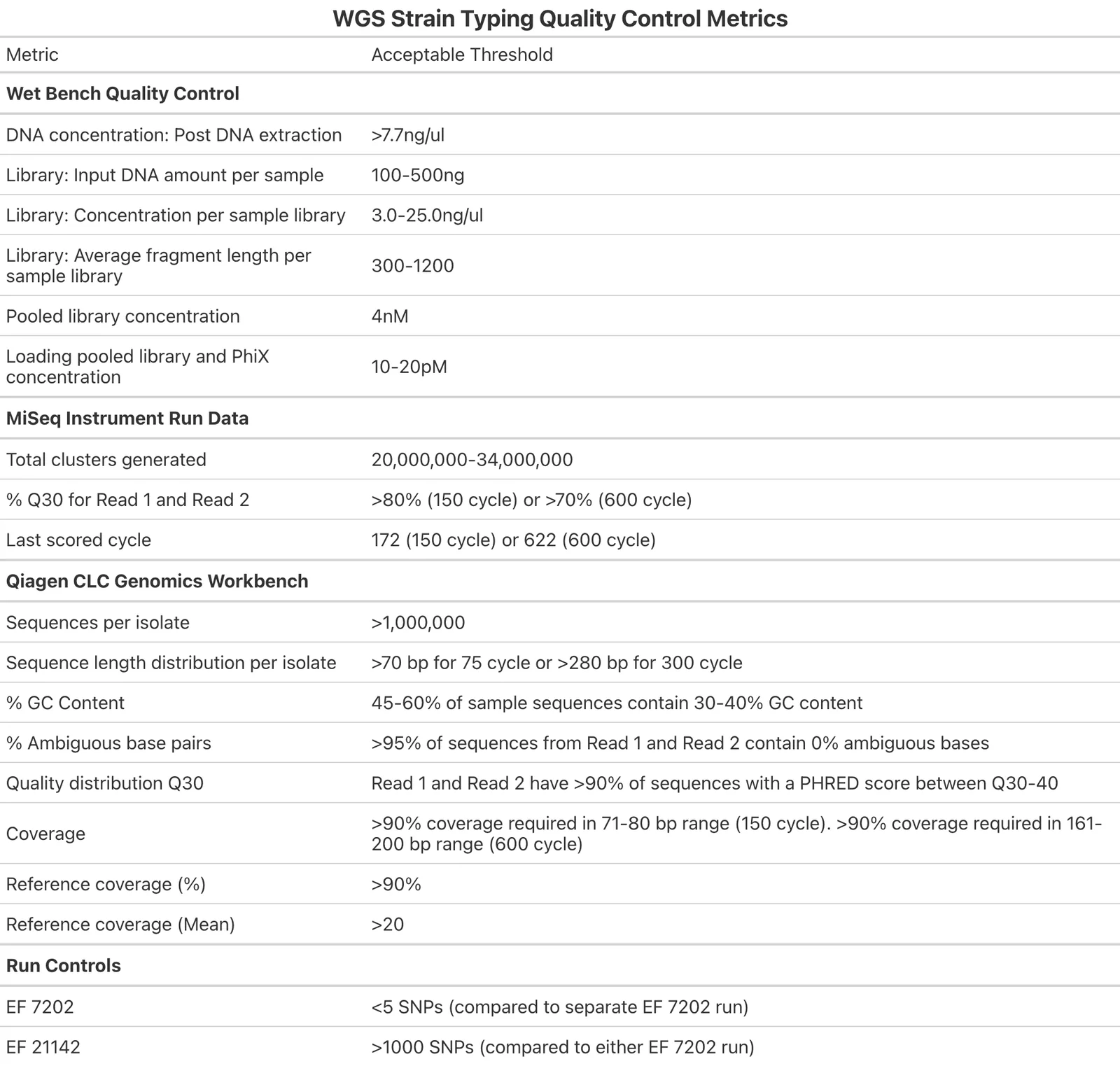
WGS SNP strain typing quality control metrics. Optimized quality control criteria for the WGS SNP pipeline. “EF 7202” and “EF 21142” refer to specific vancomycin-resistant *E. faecium* strains isolated in this study from VRE surveillance cultures.

**Fig 3:**
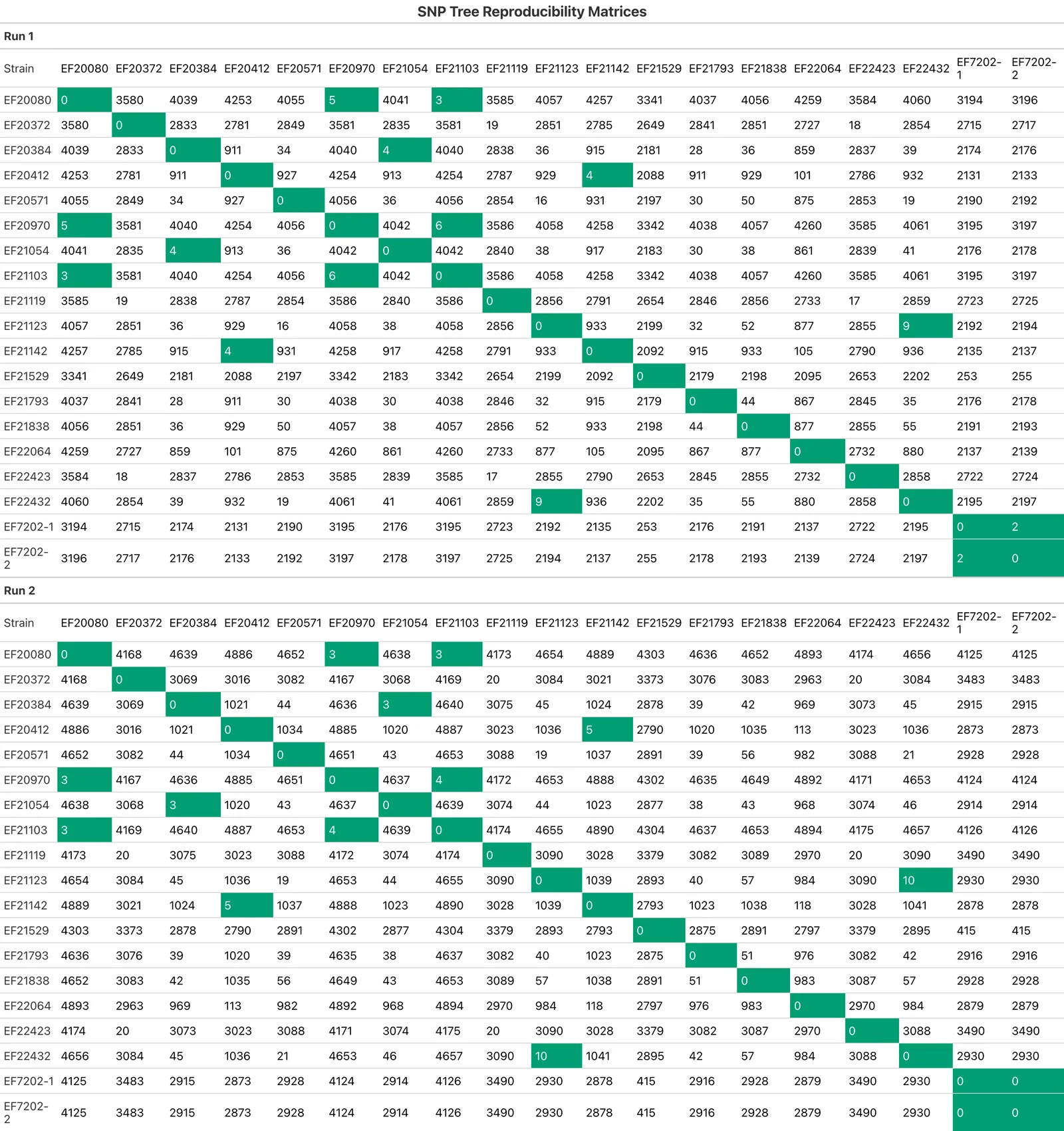
SNP tree reproducibility matrices. Two matrices are shown depicting the pairwise SNP differences between all VRE strains of a given sequencing run (e.g., “Run 1” and “Run 2”). The green highlighted cells off the long diagonal in each matrix indicate a strain comparison previously identified by PFGE as related. All of the PFGE-related strain comparisons yielded no more than ten SNP differences between runs. “EF7202-1” and “EF7202-2” refer to two different library preparations of the same VRE strain.

Toward developing a robust WGS SNP pipeline, we sought to identify quality control criteria that allowed for consistent reproducibility, as recommended by previous clinical laboratory guidelines (20, 21). Broadly, we identified the quality of DNA post-library preparation, sequencing read quality, reference strain coverage, and the use of process run controls as critical quality control features (Fig. 2). To assess the reproducibility of the entire WGS SNP pipeline, a set of 18 unique isolates consisting of both related and distinct strains (as classified by PFGE) was tested twice by two different technologists following the same sample preparation and sequence analysis procedures. With respect to identifying related strains, the two independent runs (Run 1 and Run 2, Fig. 3) demonstrated highly concordant results with no more than ten SNP deviations for a given comparison (Fig. 3, off-diagonal green cells). To further assess the precision of the WGS SNP pipeline, two different technologists performed separate sequencing runs consisting of six WGS libraries prepared from the same VREfm strain (strain EF7202, Table S2). Pairwise comparisons between these runs identified no more than one SNP for a given comparison, indicating that the quality control criteria ensured a robust assay.

### WGS SNP pipeline comparison to PFGE

**Fig 4:**
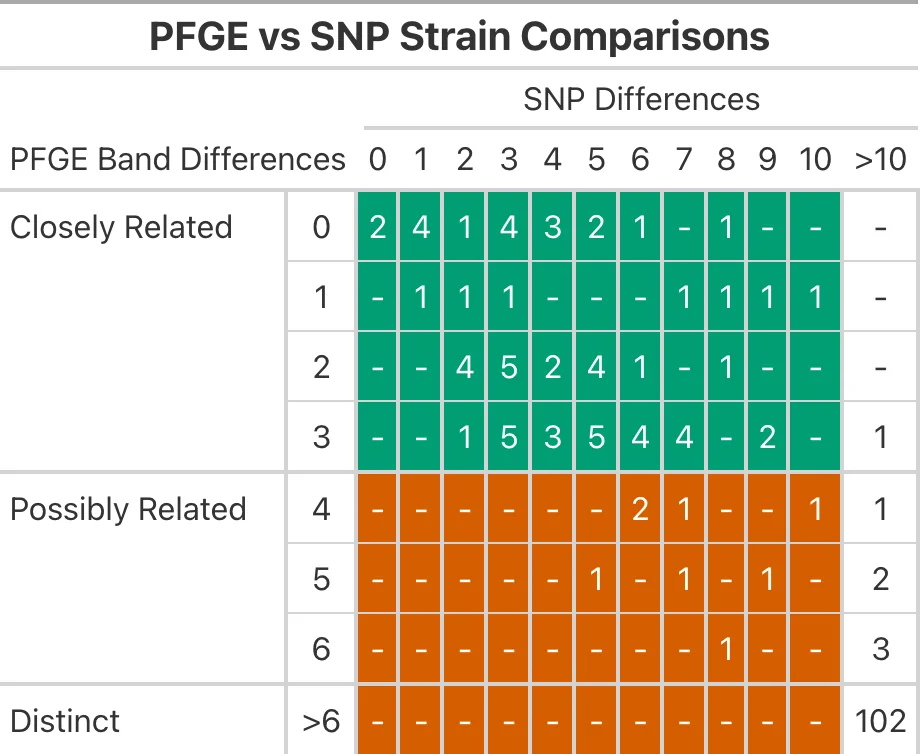
PFGE vs SNP pairwise strain comparison data. Matrix depicting both the PFGE total band differences and the SNP differences across pairwise strain comparisons, where each cell value indicates the number of pairwise comparisons. The green highlighted cells indicate the overlap between related strains as classified by PFGE and related strains as classified by WGS SNP using a threshold of <10 SNPs. Similarly, the orange highlighted cells indicate the overlap between possibly related and distinct strains, as classified by PFGE, and related strains, as classified by WGS SNP, using the same threshold.

After establishing adequate quality control criteria, the WGS SNP pipeline was tested on 58 unique VREfm isolates with known PFGE classifications from a total of 183 pairwise strain comparisons. We assessed the distribution of SNP differences between two isolates as compared to the PFGE band differences for the same pairwise comparison (Fig. 4). We found that a conservative threshold of ≤10 SNP differences for a “related strain” classification by WGS SNP yielded nearly complete concordance with the same PFGE classification (i.e., PFGE strain comparisons with ≤3 band differences). We considered ≤3 PFGE band differences as our gold standard “true positive” reference and ≥4 band differences as “true negatives,” given that ≤3 band differences correlated with the subsequent detection of epidemiological links (i.e., transmissions and outbreaks). Thus, using a threshold of ≤10 SNPs to define related strains (Fig. 4, green and orange cells) and >10 SNPs for unrelated strains, we calculated the sensitivity of WGS SNPs as 98.5% (i.e., 66 true positives divided by 67 true positives and false negatives) and the specificity as 93.1% (108 true negatives divided by 116 true negatives and false positives). Given the objective of facilitating epidemiological link detection during the course of WGS SNP surveillance, we favored high sensitivity in lieu of specificity and considered a threshold of ≤10 SNPs as a suitable criteria to trigger warranted transmission investigations.

### VREfm outbreak investigation outcomes pre- and post-WGS SNP adoption

**Fig 5:**
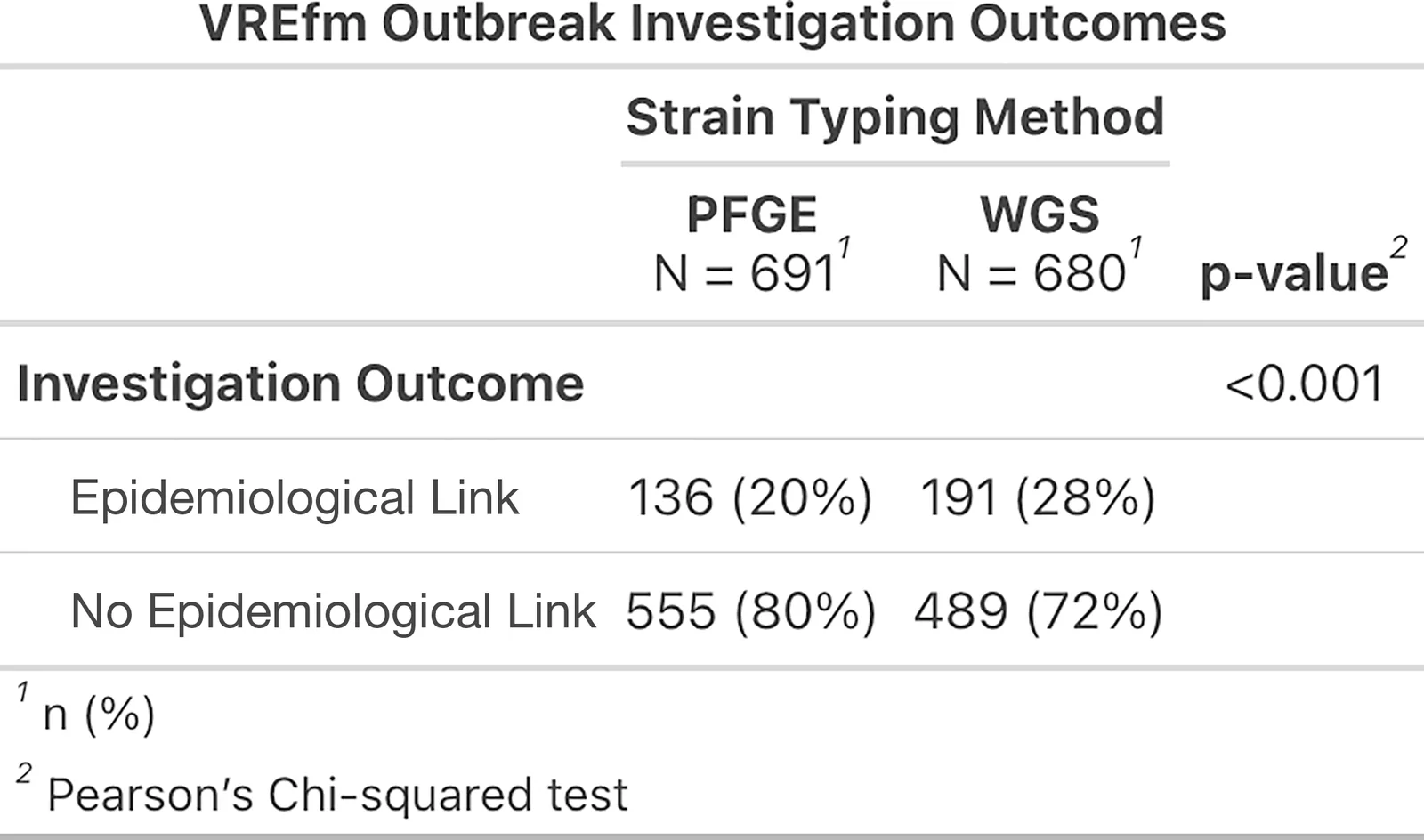
VREfm infection prevention investigation outcomes pre- and post-WGS SNP surveillance. Hospital investigation results depicting the proportion of VREfm isolates with identified epidemiological links (i.e., geo-temporal, personnel, or procedural overlaps) within the PFGE and WGS SNP strain typing cohorts. For each cohort, all VREfm isolates over the span of 1 year were investigated. The sample sizes of each cohort are indicated (“*N* =”). A Pearson’s chi-square test indicates a significant difference in the proportion of epidemiological links identified between WGS SNP and PFGE strain typing methods.

Upon completing the validation efforts, our clinical microbiology laboratory transitioned to strain typing exclusively with the WGS SNP pipeline. We sought to investigate pertinent outcomes pre- and post-WGS adoption by assessing hospital transmission and outbreak investigations by our infection prevention team during a 1-year period where PFGE was exclusively used for VREfm strain type surveillance (November 2023–2024) compared to the following year, where WGS SNP was exclusively used for strain typing (November 2024–2025). We note that a similar number of VREfm isolates were analyzed between cohorts (691 and 680 isolates in the pre- and post-WGS SNP cohorts, respectively). The results showed that a significantly greater proportion of isolates with related strain types and epidemiological links were identified in the post-WGS SNP cohort (28%, Fig. 5) compared to the pre-WGS SNP (i.e., PFGE) cohort (20%, Fig. 5) via a chi-square test. Similarly, among strain type clusters with at least one identified epidemiological link, significantly larger clusters were identified via WGS SNP as compared to PFGE (24 and 29 total strain type clusters, respectively; Fig. S1). Altogether, we interpret these results as suggesting that the WGS SNP pipeline more readily identifies related strains, facilitating the downstream identification of bona fide epidemiological links. However, we cannot distinguish between the inclusion of more false positives and the exclusion of more true positives as the chief contributor to PFGE performance from the current study design.

## DISCUSSION

In this study, we compared the performance of a WGS SNP pipeline to PFGE with respect to VREfm strain typing. Considering PFGE as the gold standard reference method, we found that the sensitivity and specificity of WGS SNP were both >90% (Fig. 4). A proportionally greater amount of isolates with related strain types and epidemiological links were identified in our hospital post-WGS SNP strain typing compared to PFGE strain typing performed the year prior (Fig. 5), suggesting that the WGS SNP pipeline improved VREfm strain typing and nosocomial surveillance compared to PFGE within a 1-year window.

We view a WGS SNP workflow as superior to PFGE for several reasons. First, the assessment of PFGE band differences is prone to user error with regard to gel alignment and relies on a potentially inconsistent qualitative interpretation between users. Strain type comparisons between separate PFGE gels are particularly difficult with respect to accurate band alignment, further limiting reproducible PFGE strain typing on a large scale. By contrast, WGS SNP allows for a high-precision and reproducible strain-typing process between users (e.g., Fig. 3; Table S2) by using pre-defined quality control criteria (Fig. 2), allowing for a quantitative basis for strain type classification. Second, the WGS SNP threshold used in our study appears to yield larger strain type clusters on average for downstream epidemiological investigation (Fig. S1). Lastly, the collection of whole-genome data allows for additional downstream applications, e.g., the detection of selection pressures and genome sweeps within clusters of local VRE isolates, which may inform hospital infection prevention practices and forecasting of outbreaks. Nonetheless, we recognize limitations of SNP-based strain typing, such as the inability to detect large genome rearrangements (known to occur among VREfm sub-clades (22) and the sensitivity of SNP detection to the choice of reference genome (16). Limitations aside, we prioritized a user-friendly bioinformatic workflow via the use of the Qiagen CLC Genomics Workbench, with the objective of maximizing reproducibility in a clinical laboratory environment and propose that, given the reproducibility achieved, WGS SNP should be the preferred choice to strain typing over PFGE if the objective is to monitor nosocomial transmissions with high resolution.

The SNP threshold used for strain typing in this study is relatively comparable to that of several recent studies whose thresholds range from ≤6 to ≤16 SNPs (6, 7, 11), albeit using different WGS SNP analysis pipelines. In these studies, SNP thresholds were established via a combination of epidemiological correlations and assessment of SNPs between multi-locus strain types. We eschewed the use of pre-defined VREfm SNP thresholds and instead leveraged the results of PFGE comparisons to identify a conservative SNP threshold specific to our data set. We view a data set-specific SNP threshold as desirable, given that VREfm strains could feasibly exhibit variable mutation rates (23, 24), which could impact the distribution of SNP differences between local isolates as compared to other geographic regions. A previous study of nosocomial *E. faecium* transmission estimated a mutation rate of seven SNPs/genome/year (10), suggesting our ≤10 SNP threshold is sufficiently relaxed to capture closely related strains within a rolling 6-week period. Nonetheless, we consider our SNP threshold as a fluid value that is subject to refinement upon the collection of more WGS SNP data with epidemiological correlations, which we view as a better surrogate of “ground truth” compared to the results obtained by PFGE. Future work will investigate the distribution of SNP differences from within-patient strain comparisons, e.g., SNP differences between isolates from separate collections of the same VREfm carrier or SNP differences between different VREfm colonies from the same isolation plate.

In summary, WGS SNP promises to be a robust approach to strain typing for the identification of nosocomial transmission involving VRE and likely other high-priority pathogens.

## Data Availability

All sequence data generated during the course of assay validation are available on the Sequence Read Archive under BioProject accession number PRJNA1455580.

## SUPPLEMENTAL MATERIAL

Supplemental material spectrum.00847-26-s0001.docx Tables S1 and S2; Fig. S1.

ASM does not own the copyrights to Supplemental Material that may be linked to, or accessed through, an article. The authors have granted ASM a non-exclusive, world-wide license to publish the Supplemental Material files. Please contact the corresponding author directly for reuse.
