# Supplementary material for: Comparing whole-genome sequencing to pulsed-field gel electrophoresis for vancomycin-resistant Enterococcus faecium hospital surveillance
Source: Microbiol Spectr. 2026 May 26;14(7):e00847-26. doi: 10.1128/spectrum.00847-26 (PMC13340300; doi:10.1128/spectrum.00847-26)
Supplement: Supplemental material — Tables S1 and S2; Fig. S1. [file spectrum.00847-26-s0001.docx]

**Table S1. Qiagen CLC Genomics Workbench Version 24 Settings**. Settings implemented in this study in the Qiagen CLC Genomics Workbench software.


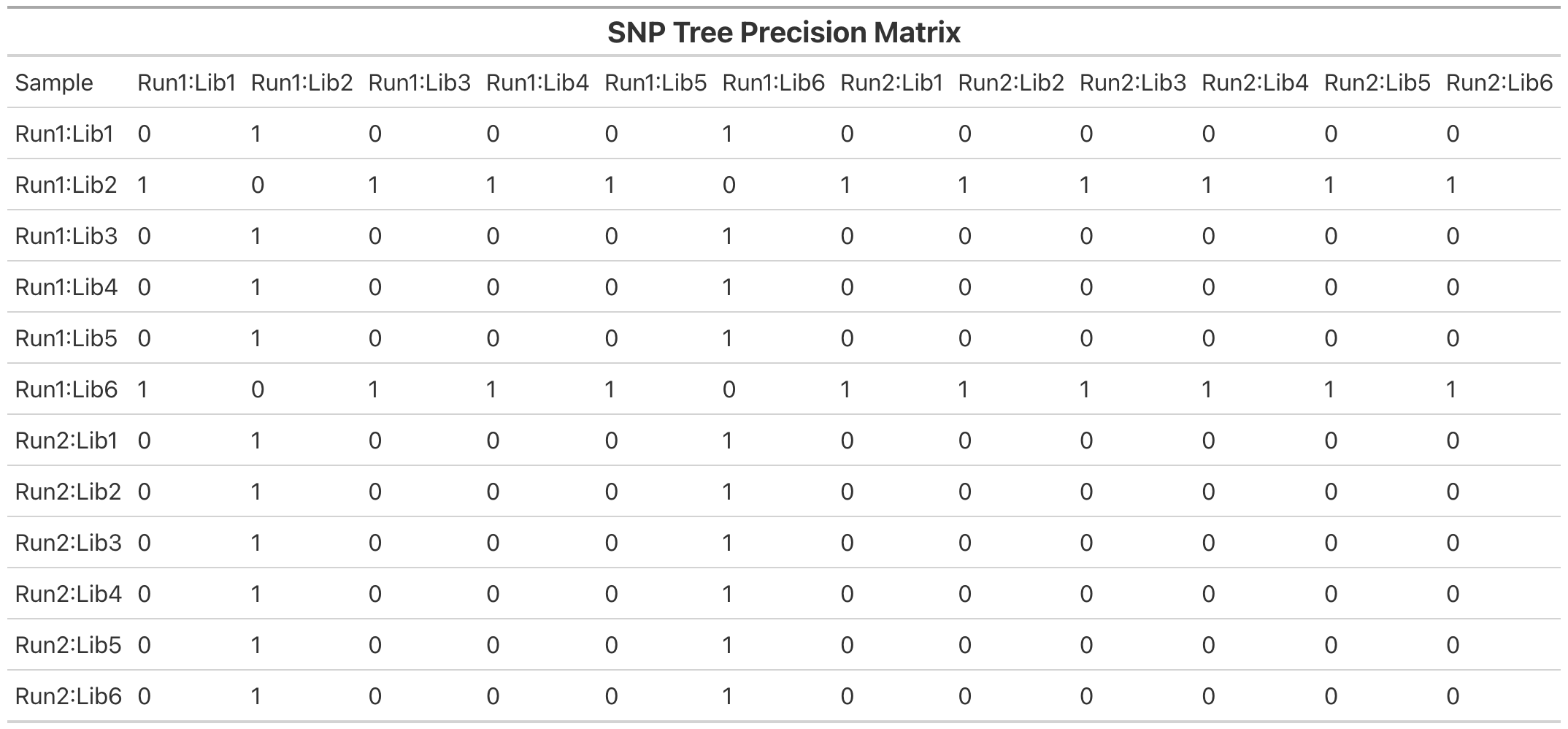


**Table S2. SNP tree precision matrix.** Matrix depicting the pairwise SNP differences between two separate runs (e.g., “Run1” and “Run2”) of six different libraries per run (e.g., “Lib1”, “Lib2”, etc.), prepared from a single VRE strain (EF 7202). Comparisons between both runs and library preparations yielded no more than 1 SNP difference.


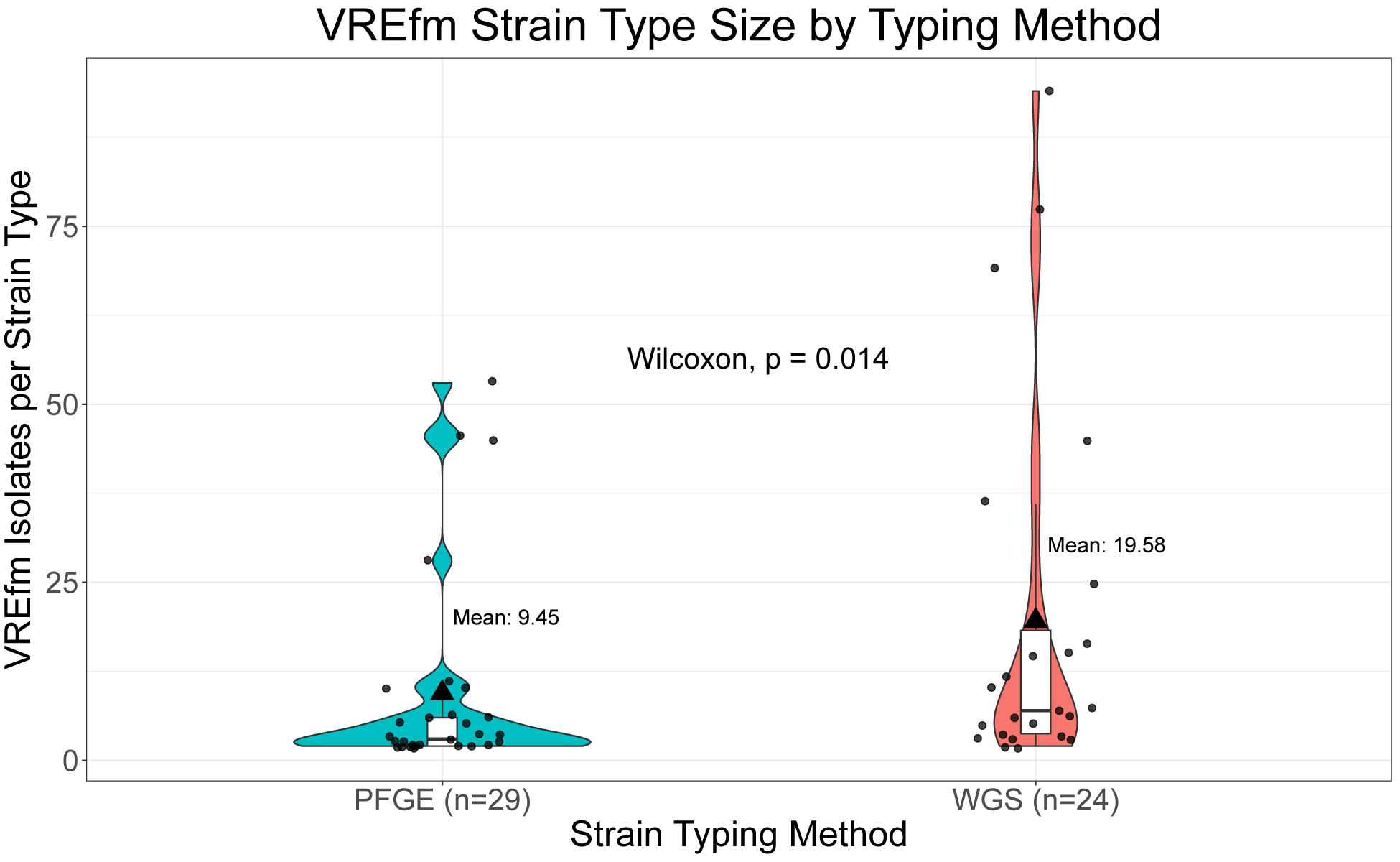


**Fig S1. VREfm Strain Type Cluster Size by Surveillance Method.** Violin and box plots depicting the distribution of strain type cluster sizes identified by PFGE or WGS SNP strain typing methods. The sample sizes (“n=”) indicate the number of unique strain type clusters identified by each method. A Mann-Whitney U test was performed to assess for a significant difference (“Wilcoxon”, p value = 0.02) between the mean of each group (black triangles) after normality tests identified a non-normal data distribution in each group. Only strain type clusters with at least one identified epidemiologic link were analyzed.
